# Modifications of lung microbiota structure in traumatic brain injury ventilated patients according to time and enteral feeding formulas: a prospective randomized study

**DOI:** 10.1186/s13054-023-04531-5

**Published:** 2023-06-21

**Authors:** A. Cotoia, R. Paradiso, G. Ferrara, G. Borriello, F. Santoro, I. Spina, L. Mirabella, K. Mariano, G. Fusco, G. Cinnella, P. Singer

**Affiliations:** 1 Department of Intensive Care, University Hospital of Foggia, Foggia, Italy; 2 Department of Animal Health, Istituto Zooprofilattico Sperimentale del Mezzogiorno, Naples, Italy; 3 Intensive Care Unit Herzlia Médical Center, Herzliya, Israel

**Keywords:** Specialized nutrition, Omega 3, Arginine, Lung microbiota, Alfa diversity, Beta diversity, VAP, Mechanical ventilation, TBI

## Abstract

**Background:**

Specialized diets enriched with immune nutrients could be an important supplement in patients (pts) with acute traumatic brain injury (TBI). Omega-3 and arginine may interact with immune response and microbiota. No data are available about the role of the specialized diets in modulating the lung microbiota, and little is known about the influence of lung microbiota structure in development of ventilator-associated pneumonia (VAP) in TBI pts. The aims of this study are to evaluate the impact of specific nutrients on the lung microbiota and the variation of lung microbiota in TBI pts developing VAP.

**Methods:**

A cohort of 31 TBI pts requiring mechanical ventilation in ICU was randomized for treatment with specialized (16pts) or standard nutrition (15pts). Alpha and beta diversity of lung microbiota were analyzed from bronco Alveolar Lavage (BAL) samples collected at admission and 7 days post-ICU admission in both groups. A further analysis was carried out on the same samples retrospectively grouped in VAP or no VAP pts.

**Results:**

None developed VAP in the first week. Thereafter, ten out of thirty-one pts developed VAP. The BAL microbiota on VAP group showed significant differences in beta diversity and *Staphylococcus* and *Acinetobacter* Genera were high*.* The specialized nutrition had influence on beta diversity that reached statistical significance only in Bray–Curtis distance.

**Conclusion:**

Our data suggest that TBI patients who developed VAP during ICU stay have different structures of BAL microbiota either at admission and at 7 days post-ICU admission, while no correlation has been observed between different enteral formulas and microbiota composition in terms of richness and evenness. These findings suggest that targeting the lung microbiota may be a promising approach for preventing infections in critically ill patients.

## Background

Enteral nutrition is indicated in critically ill patients mechanically ventilated in Intensive Care Unit (ICU), due to their catabolic and stress states [1]

In patients with acute traumatic brain injury (TBI), the use of specialized diets enriched with immune nutrients such as omega-3 polyunsaturated fatty acids and arginine could be an important supplement [2, 3]. These particular nutrients may improve immune responses and [4] have modulating effect on gut microbiota [2, 3, 5]. TBI influences gut microbiota composition whose alterations may regulate a proinflammatory response aggravating secondary brain injury and functional outcome due to the presence of “gut-brain axis” [6].

Recent studies have highlighted the presence of a “gut-lung axis,” since alterations of intestinal microbiota communities may have profound effect on lung diseases [7]. Compared with the gut, the healthy lungs present eubiosis with a more hostile environment for the growth of bacteria due to bidirectional movement generating by the air-flow, a lipid-rich surfactant coating of alveoli, poor nutrients quantities and an aerobic environment [8, 9].

In a plethora of lung diseases, the microbiota changes drastically, determining the dysbiosis [10] and the upregulation of inflammatory signals [10]. Furthermore, in critically ill ICU patients sedatives and endotracheal intubation decrease the mucociliary clearance and cough reflex, leading to a thriving microbiota [11, 12].

Dysbiosis has been recently investigated in mechanically ventilated patients with ventilator-associated pneumonia (VAP) or sepsis, but little is known about the influence of lung microbiota composition in development of VAP in ICU patients. Furthermore, no data are available about the role of the nutrition enriched specific nutrients in modulating the lung microbiota [10].

For these reasons, the aims of this study are to evaluate the impact of specific nutrients on the lung microbiota and the variation of normal respiratory microbiota in TBI patients developing VAP in ICU.

## Materials and methods

### Patients

After approval of the local research Ethics Committee (Comitato Etico of Ospedali Riuniti, Foggia, Italy, 46/CE/2022), written informed consent was obtained by each patient or by relatives. The study was performed from February 2021 to March 2022 at the Department of Intensive Care, University Hospital of Foggia, Italy (ClinicalTrials.gov: NCT05854264).

Inclusion criteria were: adults (age > 18 years), TBI patients requiring mechanical ventilation. Exclusion criteria were: pregnant women, patients with gastric content inhalation (and possible aspiration pneumonia), chronic inflammatory bowel diseases, major abdominal surgery and/or organs/tissues transplant, patients on corticosteroid and/or immunosuppressive therapy.

Patients were randomized in two groups: a standard enteral feeding or a specialized formula enriched in fish oil and arginine. Standard formula contained 1 kcal/ml, fat 30%, carbohydrate 55%, protein 15%, while specialized nutrition formula contained 1.01 kcal/ml, fat 25%, carbohydrate 53%, protein 22% enriched with omega 3 330 mg/100 ml and arginine 1.3 g/100 ml. Randomization was performed by computer-generated random allocation sequence by simple randomization. The calories (25 kcal/kg/day) and protein (1.2 g/kg/day) target were progressively reached in the first week of ICU stay for all patients.

### Data and samples collection

At ICU admission, we collected demographic, clinical, laboratory data, Simplified Acute Physiology Score (SAPS) II, Acute Physiologic Assessment and Chronic Health Evaluation (APACHE) II Score, mNutric score, Marshall score. Laboratory, clinical and treatment data were also collected. Moreover, ICU length of stay and outcome were recorded. VAP was diagnosed using consensus criteria [13]. Bacteria cultural tests were performed at admission, every week and whenever needed in addition to the active surveillance. Empiric antibiotic treatment was evaluated for associations with clinical–demographic data, while its changes were evaluated based on antibiograms [14]. Furthermore, Bronco Alveolar Lavage (BAL) samples were collected at admission in ICU (T0) and at day 7 (T7) for the microbiota analysis. Clinical follow-up at day 28 was obtained.

### BAL samples analysis

For each lavage sample of the upper and lower airway, 20 ml of sterile isotonic saline solution were instilled with subsequent gentle suctioning. As soon as the upper and lower airway lavage samples were collected, they were centrifuged (11,000 g for 10 min). Subsequently, the pellet was collected and stored at − 80 °C until analysis. Defrosted samples were centrifuged a second time (22,500 g for 30 min), and the resulting pellet was used for DNA isolation by the DNeasy PowerSoil Kit (QIAGEN) according to manufacturer’s instructions. Of the 62 BAL samples, one sample belonging to the standard nutrition group (ID #16 at T7) was excluded by the study because it resulted damaged at the opening. An aliquot of the sterile saline used in sample collection and processing was included in the set of analyzed samples as a negative extraction control. It was processed and sequenced along with all the collected samples, and the identified features were evaluated in order to exclude potential sources of contamination. Extracted DNA was quantified using a high-sensitivity Qubit™ fluorometer.

### Amplification and sequencing

The microbiota of the 61 BAL samples included in the study was characterized by high throughput sequencing of the 16S rRNA gene. For this purpose, the 16S Ion Metagenomics kit (Life Technologies) was used, following the manufacturer’s instructions. Briefly, this method includes two separate PCR reactions, amplifying, respectively, V2-4–8 and V3-6, V7-9 regions. Each PCR reaction was carried out on 5 ng of microbial DNA. The thermal profile for both PCR reactions consisted of 1 cycle at 95 °C for 10 min, 30 cycles consisting of 95 °C for 30 s, 58 °C for 30 s, 72 °C for 20 s, and a final extension cycle at 72 °C for 7 min. After amplification, PCR products were purified using the Agencourt AMPure beads (Beckman Coulter Inc, Atlanta, Georgia), eluted in Low TE buffer and quantified by the Qubit dsDNA HS Assay kit (Life Technologies). After amplification, a pool of 100 ng of total DNA for each sample (50 ng from each reaction) was used for library preparation. Libraries were barcoded using Ion Xpress Barcodes Adapters (Life Technologies) and amplified in an emulsion PCR on the Ion Chef system (Life Technologies) according to the manufacturer’s instructions. Sequencing was performed on the Ion S5 System (ThermoFisher) using the Ion 330 Chip kit (Life Technologies). The raw sequences were submitted to NCBI under BioProject accession number PRJNA892871.

### Data analysis

After sequencing, reads were pre-processed for quality control by the DADA2 software to denoise, remove primers, de-replicate single-end sequences, remove chimaeras and exclude low quality reads [15]. Filtered and de-noised reads were therefore trimmed at 200 bases and resolved to high-resolution Amplicon Sequence Variants (ASVs), representing the inferred original biological sequences [16]. The QIIME 2–2020.2 software was used for the downstream taxonomic analysis at phylum, family and genus level. Representative ASVs sequences were processed for multiple sequence alignment using MAFFT software [17]. FastTree[18] was then used to infer unrooted and subsequently rooted maximum-likelihood phylogenetic trees representing the phylogenetic relatedness of ASVs (QIIME2 phylogeny align-to-tree-mafft-fasttree plugin). ASVs were taxonomically classified using the QIIME2 feature-classifier classify-sklearn plugin, which is a Naïve Bayes classifier pre-trained on SILVA 138 database, including reference sequences clustered at 99% similarity [19, 20]. All samples resulted in more than 15,000 sequences and were therefore suitable for subsequent analysis.

The most abundant features identified in the negative control were matched with those found in the analyzed BAL samples to exclude the presence of any contamination able to critically impact results [21]. All the features found in the negative control were either absent or present at a relative frequency value lower than 2% in the BAL samples. Based on this evidence and the best practices for analyzing microbiomes [21] we decided to filter out low abundance ASVs by removing all the features with a minimum frequency lower than 200.

Data were first evaluated by the Kolmogorov–Smirnov test to verify their non-normal distribution. Following taxonomic analysis, differences in alpha and beta diversity for unpaired samples (standard vs. specialized nutrition at T7) were analyzed by using the QIIME 2 software. Alpha diversity analysis was based on the observed features (a measure of species richness based on ASVs abundance) and Shannon (a quantitative measure of both the number of species and the inequality between species abundances) indexes and was carried out using the Mann–Whitney test for unpaired samples. Beta diversity Principal Coordinates Analyses (PCoA) was also performed between the two nutrition groups using Bray–Curtis (a quantitative measure of community dissimilarity), unweighted (a qualitative measure of community dissimilarity incorporating phylogenetic relationships between the features) and weighted (a quantitative measure of community dissimilarity incorporating phylogenetic relationships between the features) UniFrac distances matrices by the QIIME 2 software [22]. Alpha and beta diversity analysis were performed between paired samples (T0 vs. T7) by using a specific tool of QIIME 2 software for paired samples (http://github.com/qiime2/q2-longitudinal) [23].

Compositional beta diversity analysis was performed by Robust Aitchison Principal Component Analysis with DEICODE through QIIME 2 software. This principal component analysis (PCA) is based on a compositional distance metric which can properly account for the relative changes of microbial taxa abundances. The Robust Aitchison PCA weights more heavily microbes displaying large fold changes across samples and can handle sparse data (zero values) through the use of matrix completion. It can also provide information on the taxonomic abundance changes responsible for sample clustering and can therefore identify the taxa driving the difference between sample groups [24].

### Sample size and statistical analysis

Sample size calculation was estimated by performing a statistical power analysis [25]. Based on this calculation, 16 patients for each group will be enough to provide a 95% power for a two-sided error of 5% to show any difference in microbiota diversity***.*** Under the assumption that some patients would be excluded for various reasons, we planned a total of 40 patients.

The normality of distribution was assessed by the Shapiro–Wilk test or Kolmogorov–Smirnov test. Unpaired Student T-test or Mann–Whitney U-test were used for normally or not normally distributed data, respectively. Differences between the groups at each time point were examined post hoc using an independent sample t-test or Chi-square test. Bray–Curtis, unweighted, weighted and UniFrac distances matrix, Bokulich longitudinal, Robust Aitchison Principal Component Analysis with DEICODE and Principal Coordinates Analyses (PCoA) were performed using QIIME 2 software [22]. A value of *p* < 0.05 was considered statistically significant.

## Results

### Patients

**Fig. 1:**
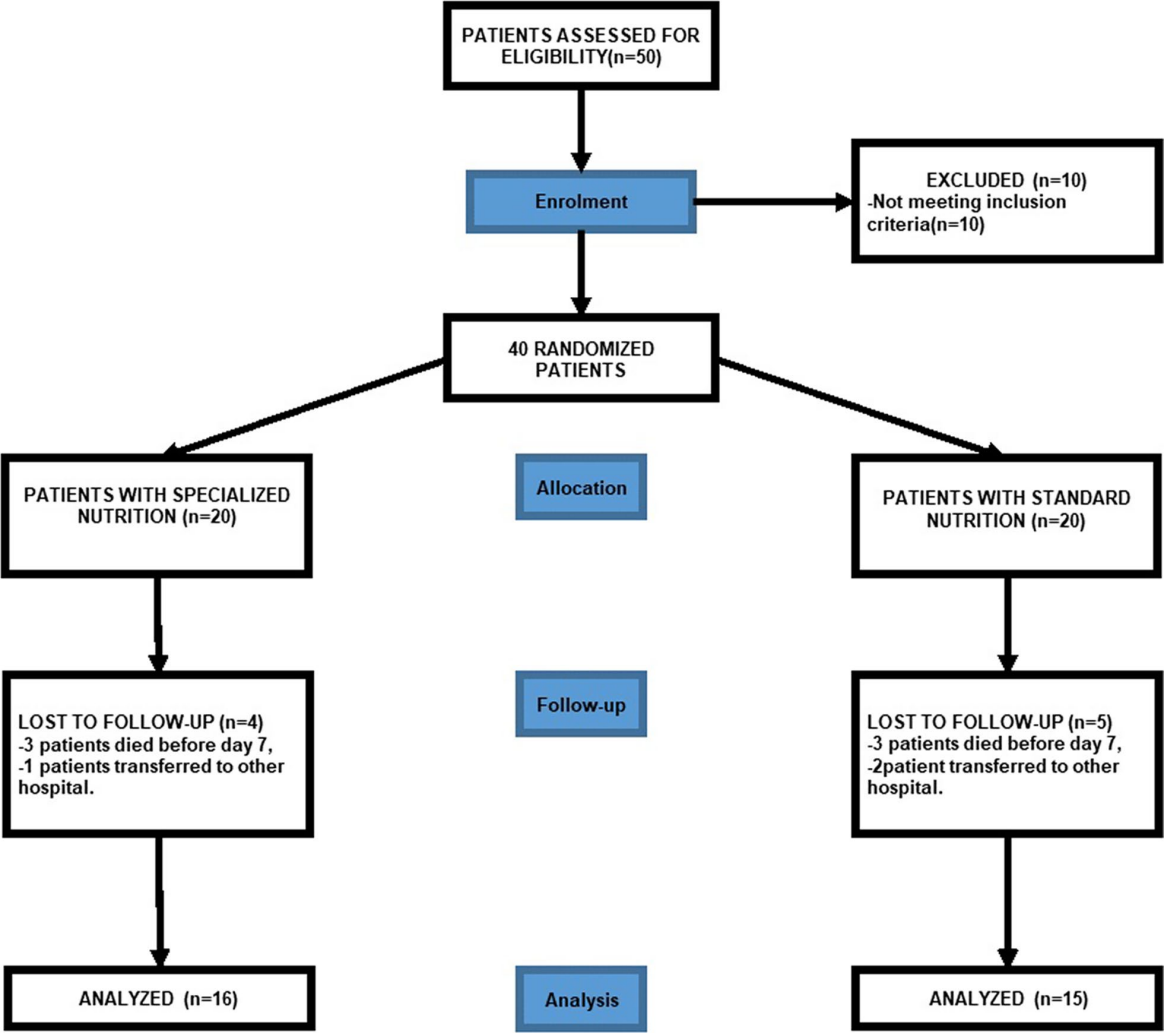
Flowchart representing the patients cohort selected for this study

Fifty patients were screened for eligibility. Ten out of fifty patients were not eligible because they did not meet inclusion criteria. Nine patients were also excluded from the study because they either died before day 7 or were transferred to other hospitals. Thirty-one patients were enrolled and divided into two groups: Standard Nutrition Group (*N* = 15) and Specialized Nutrition Group (*N* = 16; Fig. 1). All patients received 25 kcal/kg/day and 1.2 gr/day of protein intake within one week from admission and until their discharge from the ICU. No patient reported any intolerance, hypersensitivity or side effects related to enteral treatment. No difference was found in age, gender and weight of patients’ characteristics between the two groups (Table 1). No significant differences were found either in SAPS or APACHE II score (Table 1). Lymphocyte counts at day 7 was (1.07*10^3/ul ± 0.55) in specialized Nutrition and 0.91*10^3/ul ± 0.65 in Standard nutrition (*p* value = 0.25). Furthermore, mechanical ventilation days, ICU length of stay and 28-day mortality were similar in both specialized and standard nutrition groups (Table 2).

**Table 1:** Demographic, clinical and laboratory data at the admission in ICU (T0)

	Standard nutrition (*N* = 15)	Specialized nutrition (*N* = 16)	*p*-value
Sex (M: F)	11: 4	13: 3	0.433
Age (Years)	56 ± 22	51 ± 22	0.56
GCS	7 [7, 8]	7 [6.75–7.25]	0.69
BMI (Kg/m2)	25.3 ± 4.3	24.5 ± 3	0.56
mNUTRIC score	6 [4–6.5]	4 [3–5.25]	0.34
APACHE II score	20 [18–23]	17.5 [13.75–23]	0.28
SAPS II score	53 [49–58.5]	46.5 [40–59.25]	0.22
Albumin (g/dl)	3.48 ± 0.66	3.22 ± 1.1	0.94
Proteins (g/dl)	5.41 ± 0.5	5.63 ± 1.2	0.48
White blood cells (10^3/mcl)	13.5 ± 3.7	12 ± 4.7	0.57
Procalcitonin (ng/ml)	0.5 ± 1	0.2 ± 0.6	0.31
Platelets (10^3/mcl)	214.2 ± 70.2	196.37 ± 83.7	0.37
Platelet distribution width (fL)	12.3 ± 1.8	13.6 ± 3.1	0.16
Mean platelet volume (fL)	10.7 ± 0.8	11.1 ± 1.4	0.26
Plateletcrit (%)	0.2 ± 0.006	0.2 ± 0.007	0.22
Creatinine (mg/dl)	1.5 ± 1.8	1.6 ± 2	0.22
Vasopressor use, *N*(%)	10 (67%)	12 (75%)	0.43
smokers,*N*(%)	4 (26%)	7 (43%)	0.10
Marshall score	2 [2, 3]	2.5 [2, 3]	0.59
*Comorbidities*			
Neurological	5 (33%)	3 (19%)	0.19
Cardiovascular	9 (60%)	6 (37%)	0.05
Diabetes	2 (13%)	0 (0%)	0.10
Respiratory	2 (13%)	1 (6%)	0.36
Empiric cephalosporin treatment at T7, *N* (%)	7 (47%)	5 (31%)	0.19

Data were expressed as mean ± SD, median [25–75th percentile] or %

**Table 2:** Table representing ICU data and outcome of patients

	Standard nutrition (*N* = 15)	Specialized nutrition (*N* = 16)	*p*-value
Tracheostomy, *N*	11 (73%)	11 (69%)	0.61
Days of intubation before tracheostomy (dd)	11 ± 3.5	13 ± 3	0.07
Invasive ventilation (dd)	17.5 ± 5.1	13.1 ± 4.4	0.11
ICU Los (dd)	19.5 ± 7.6	14.5 ± 3.7	0.25
ICU 28-daymortality, *N* (%)	7 (47%)	4 (25%)	0.06

Data were expressed as mean ± SD or %

### BAL microbiota at ICU admission

**Fig. 2:**
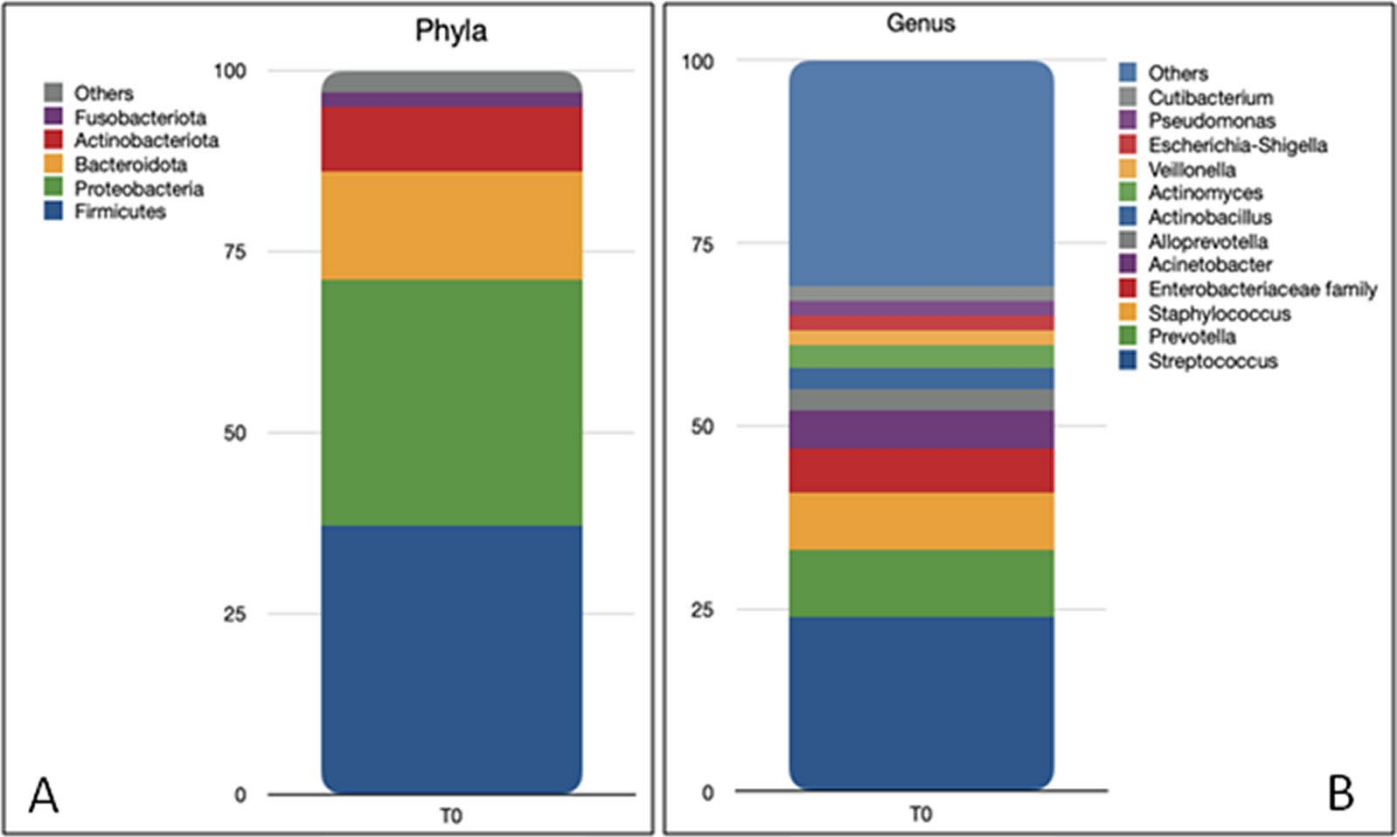
Taxa bar plots showing the most representative (more than 2%) Phyla **A** and genera **B** composing the BAL microbiota of the patients at ICU admission identified by 16S rRNA amplicon sequencing

Sequencing data after reads pre-processing of the 61 BAL samples included in the study displayed a total number of sequences of 15,752,239, with a mean number of sequences of 225,679 per sample in the standard nutrition group (median number: 233,465 sequences, minimum number: 35,848 and maximum number: 409,927) and 287,735 in the immune nutrition group (median number: 251,538, minimum number: 16,599 and maximum number: 1,186,132). At T0, the taxonomic analysis of samples collected from all patients exhibited the presence of 19 Phyla (Fig. 2A). The most abundant (with mean relative frequencies > 2%) were Firmicutes (37.17% ± 21.24%), Proteobacteria (34.48% ± 22.45%), Bacteroidetes (14.74% ± 16.45%) and Actinobacteria (8.92% ± 7.06%).

At the Family level, the most abundant were *Streptococcaceae* (20.81% ± 17.95%), *Prevotellaceae* (12.34% ± 14.6%), *Pasteurellaceae* (9.74% ± 16.07%), *Staphylococcaceae*(6.66% ± 17.34%), *Enterobacteriaceae*(5.35% ± 15.84%), *Moraxellaceae*(4.77% ± 7.84%), *Pseudomonadaceae.*

(3.94% ± 8.4%), *Neisseriaceae* (2.63% ± 5.41%), *Micrococcaceae*(2.49% ± 2.95%), *Veillonellaceae* (2.33% ± 3.24%).

At the Genus level, a total of 339 genera were observed (Fig. 2B), with the most frequent represented by *Streptococcus* (20.77% ± 17.94%), *Prevotella* (9.05% ± 12.74%), *Staphylococcus* (6.64% ± 17.35%), *Acinetobacter* (4.22% ± 6.97%), an unclassified genus of the family of *Enterobacteriaceae*(3.98% ± 10.70%),*Pseudomonas*(3.93% ± 8.40%),*Actinobacillus*(3.55% ± 10.31%), *Haemophilus* (3.17% ± 7.99%), *Alloprevotella* (3.09% ± 4.50%), and an unclassified genus of the family *Pasteurellaceae* (2.81% ± 5.25%), *Neisseria* (2.41% ± 5.29%) and *Veillonella* (2.07% ± 3.00%).

Analysis of alpha diversity (i.e., the measure of microbiota diversity and richness) and beta diversity (i.e., the measure of similarity or dissimilarity of two communities) showed no difference according to gender (male vs female), age (18–29 years – young adult vs 30–59 years—middle adult vs > 60 old adult), BMI (normal-weight vs overweight vs obesity grade I), smoking (smokers vs non-smokers) and Marshall score (score 2 vs score 3) (Table 3), with the only exception observed for a unique metric, the Unweighted UniFrac distance matrix, calculated according to gender (*P* = 0.008). The same analyses were also carried out comparing the two nutrition groups at T0, displaying no significant differences between the two groups for any of the investigated indexes (Table 3).

**Table 3:** Analysis of Alpha and Beta diversity in patients

	Alpha—diversity (Kruskal–Wallis) *P* value		Beta—diversity (Permanova) *P* value		
	Observed_features	Shannon_entropy	Bray_Curtis	Weighted_Unifrac	Unweighted_Unifrac
Gender	0.136	0.114	0.176	0.209	0.008
Age	0.432	0.818	0.477	0.868	0.132
BMI	0.760	0.749	0.568	0.574	0.701
Smoking	0.827	0.965	0.987	0.872	0.933
Marshall score	0.765	0.266	0.939	0.635	0.555
Nutrition	1.000	0.579	0.178	0.319	0.177

*P*-values obtained from alpha and beta diversity analysis carried out on BAL samples collected from patients at T0

### BAL microbiota at day 7 after hospitalization

#### Standard vs specialized nutrition

At T7, intragroup analysis showed no differences *vs* T0 for either alpha diversity indexes (Wilcoxon signed-rank test; Observed features and Shannon index; *P* > 0.1) and beta diversity distances (Kruskal–Wallis test; Bray–Curtis, Unweighted and Weighted UniFrac matrixes; *P* > 0.08).

Intergroup analysis of alpha diversity at T7 displayed no differences both in the microbial richness (Kruskal–Wallis test; Observed features index; *H* = 1.555; *P* = 0.212) and evenness (Kruskal–Wallis test; Shannon index; *H* = 1.453; *P* = 0.227), indicating that the specialized nutrition did not significantly affect the richness of microbial taxa. Indeed, both groups exhibited comparable levels of observed ASVs, with 280 mean number of ASVs ± 92.7 SD in the specialized nutrition group, vs. 319.5 ± 116.1 in the standard nutrition group.

**Fig. 3:**
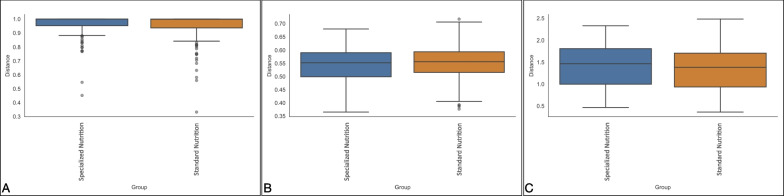
Beta diversity analysis of BAL microbiota at T7. Distance boxplots showing standard nutrition (blue) and specialized (orange) groups. Bray–Curtis, *P* = 0.013 **A**, unweighted UniFrac, *P* = 0.738 **B** and weighted UniFrac, *P* = 0.104 **C** distances

The beta diversity of the microbiota of the two diet-based groups was evaluated comparing the intergroup distances by both taxonomic and phylogenetic approaches. Measures of species abundance (PERMANOVA; Bray–Curtis; *pseudo-F* = 4.66*;* *P* = 0.013; Fig. 3A), presence (PERMANOVA; unweighted UniFrac; *pseudo-F* = 0.097*;* *P* = 0.738; Fig. 3B) and both presence and abundance considered together (PERMANOVA; weighted UniFrac; *pseudo-F* = 3.037*;* *P* = 0.104; Fig. 3C) confirmed that the type of nutrition had low influence on microbiota diversity, that reached statistical significance only in the Bray–Curtis distance, suggesting differences only in the relative abundance of shared ASVs between groups.

**Fig. 4:**
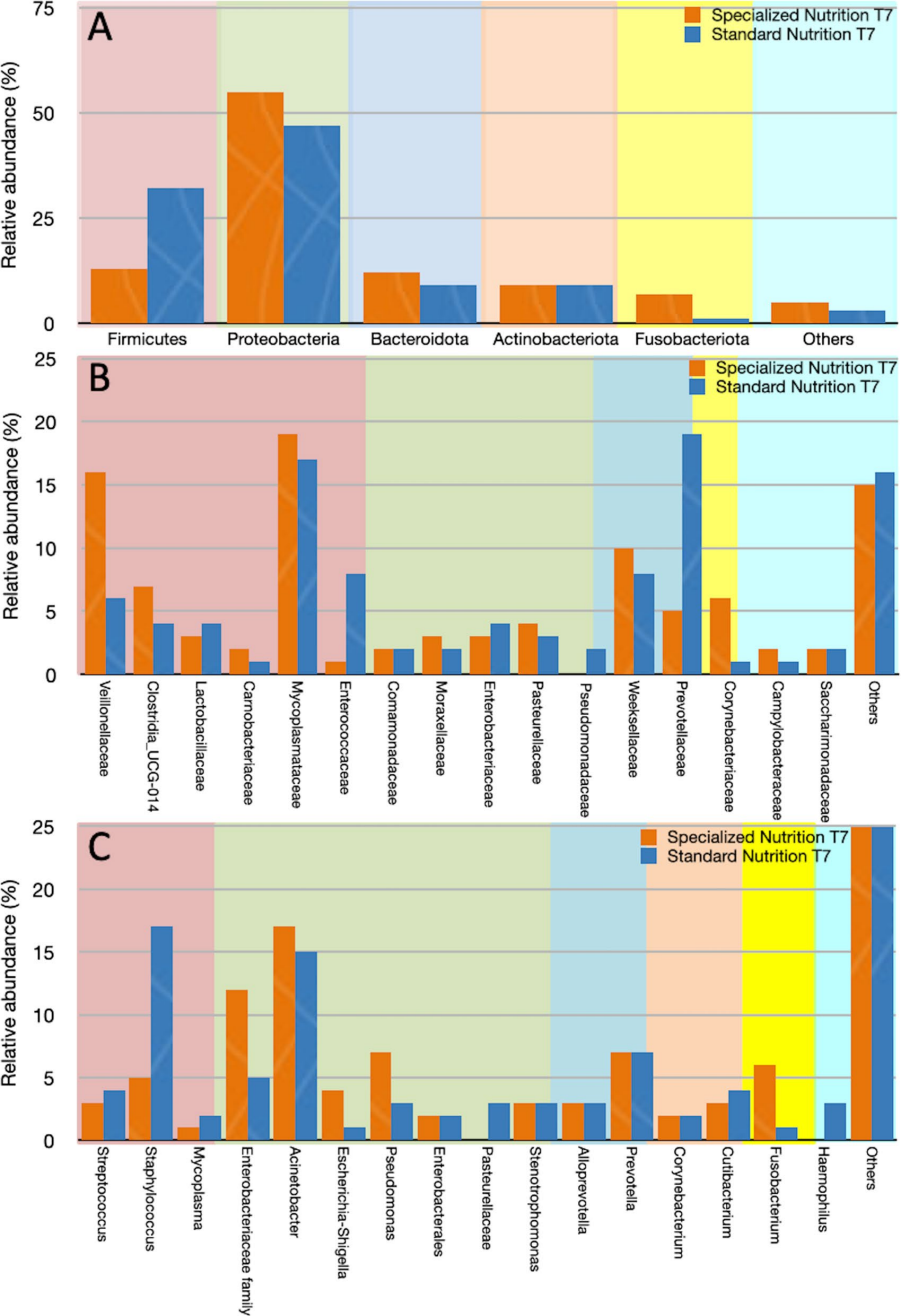
Comparison of taxa relative frequencies between the specialized nutrition (orange) and standard nutrition (blue) groups at the Phyla **A**, Family **B** and Genus **C** levels

These data are consistent with the results of the taxonomic analysis at T7, which showed the presence of comparable numbers of Phyla, Families and Genera between the two diet groups with different values of relative frequencies (Fig. 4).

#### VAP vs no VAP

No patient developed pulmonary infections during the first seven days, while 10 patients developed VAP over 14 days after ICU admission (Table 4). The microbiological and 16S sequencing data appeared well correlated to each other, with the exception of those related to *Klebsiella* sp., which was not found by 16S sequencing analysis in any of the samples collected from the VAP patients who exhibited the presence of this microorganism by cultural method. However, among all the analyzed samples, the 16S sequencing method was able to identify *Klebsiella* spp. in 6 samples collected from patients in both diet groups even if at low frequency values, lower than 1.5% (data not shown), thus confirming the ability of this method to identify this microorganism. Thereafter, over 14 days after ICU admission ten patients developed VAP, equally distributed between the two nutrition groups (5 out of 15 in the standard nutrition group and 5 out 16 in the special nutrition group) (Table 4). This evidence suggests that special nutrition does not play a protective role in preventing VAP.

**Table 4:** Patient who developed VAP during ICU stay

Patient ID	Infection > 14 days	Infection > 21 days	Infection > 28 days	BAL culture	Relative abundance (%) at T0 by 16S	Relative abundance (%) at 7 days by 16S
17	+	+	+	Klebsiella pneumoniae	n.f.*	n.f
18	+	+	+	Staphylococcus aureus Klebsiella pneumoniae	49.1 n.f	47.7 n.f
21	0	+	+	Pseudomonas aeruginosa Staphylococcus aureus	0.8 12.9	0.4 43.8
**23**	**0**	** + **	** + **	**Staphylococcus aureus** **Acinetobacter baumannii** **Klebsiella pneumoniae**	**3.4** **31.6** **n.f**	**8.0** **85.7** **n.f**
**24**	**0**	** + **	** + **	**Acinetobacter baumannii**	**9.1**	**0.3**
**25**	**0**	** + **	** + **	**Pseudomonas aeruginosa Acinetobacter baumannii**	**0.3** **1.0**	**3.2** **8.7**
**26**	**0**	** + **	** + **	**Klebsiella oxytoca**	**n.f**	**n.f**
**27**	**0**	** + **	** + **	**Klebsiella pneumoniae**	**n.f**	**n.f**
28	0	0	+	Staphylococcus aureus	0.01	0.03
31	+	0	0	Klebsiella pneumoniae	n.f	n.f

In bold treated with specialized nutrition, the others were treated with standard formula

^*^n.f.  Not found

In order to evaluate the presence of eventual differences in microbiota composition between VAP and non-VAP patients, they were regrouped in VAP (median time of VAP development after ICU admission: 15 [12.5 – 18.75] days) vs no VAP and alpha and beta diversity analysis was repeated independently from the nutrition treatment.

**Fig. 5:**
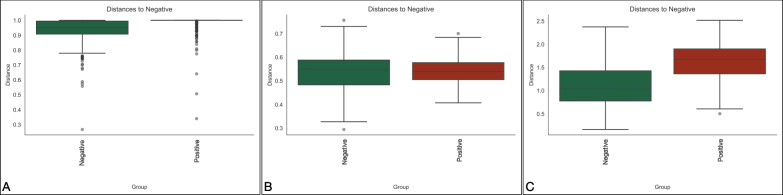
beta diversity analysis of BAL microbiota at T7 between pneumonia and no pneumonia groups. Distance boxplots showing negative (green) and positive (red) groups. Bray–Curtis, *P* = 0.002 **A** unweighted UniFrac, *P* = 0.004 **B** and weighted UniFrac, *P* = 0.002 **C** distances

At T7, Alpha diversity analysis (Kruskal–Wallis test; Observed features index; *H* = 0.970; *P* = 0.324; Kruskal–Wallis test; Shannon index; *H* = 0.879; *P* = 0.348) indicated that microbial richness and evenness were similar in patients that developed VAP vs patients that did not, while beta diversity analysis was different (Fig. 5) for all the three considered distances (PERMANOVA; Bray–Curtis; *pseudo-F* = 2.456*;* *P* = 0.002; unweighted UniFrac; *pseudo-F* = 2.399*;* *P* = 0.004: weighted UniFrac; *pseudo-F* = 7.363*;* *P* = 0.002), thus suggesting that different structures of BAL microbiota might determine clinical outcomes during hospitalization.

**Fig. 6:**
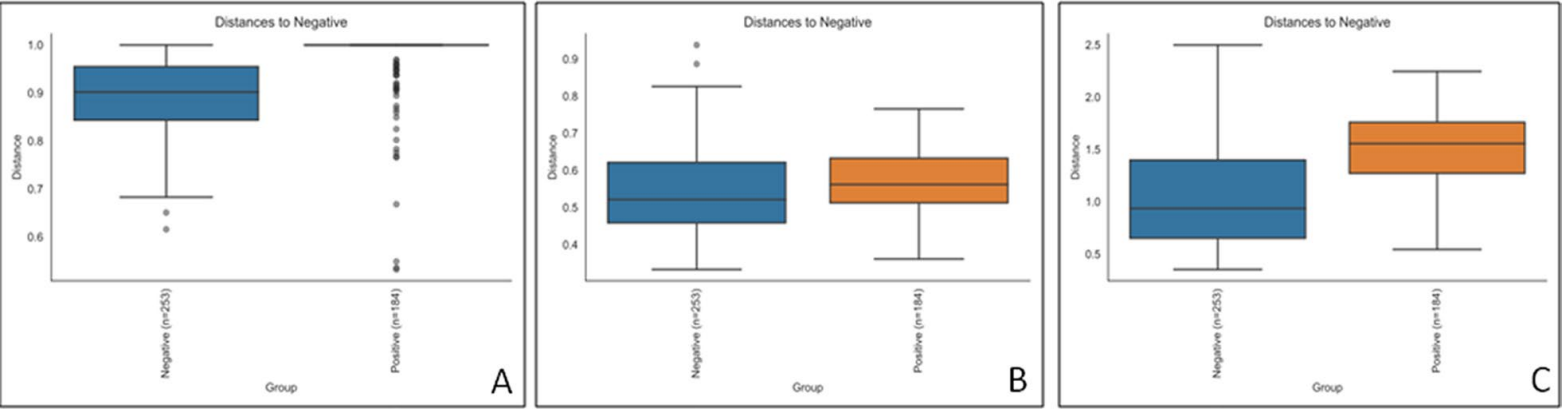
beta diversity of BAL microbiota at T0 between the two VAP-status groups. Distance boxplots showing negative (blue) and positive (orange) groups. Bray–Curtis, *P* = 0.001 **A** unweighted UniFrac, *P* = 0.001 **B** and weighted UniFrac, *P* = 0.001 **C** distances

Furthermore, we observed the same significance on T0 between VAP and no VAP groups for alpha (Kruskal–Wallis test; Observed features index; *H* = 0.147; *P* value = 0.705; Kruskal–Wallis test; Shannon index; *H* = 0.02; *P* value = 0.963) and beta (PERMANOVA; Bray–Curtis; *pseudo-F* = 3.026*;* P = 0.001; unweighted UniFrac; *pseudo-F* = 2.801*;* *P* = 0.001: weighted UniFrac; *pseudo-F* = 10.544*;* *P* = 0.001) diversity analysis (Fig. 6), supporting the evidence that the possibility to develop respiratory infections during hospitalization might be associated with the structure of BAL microbiota.

**Fig. 7:**
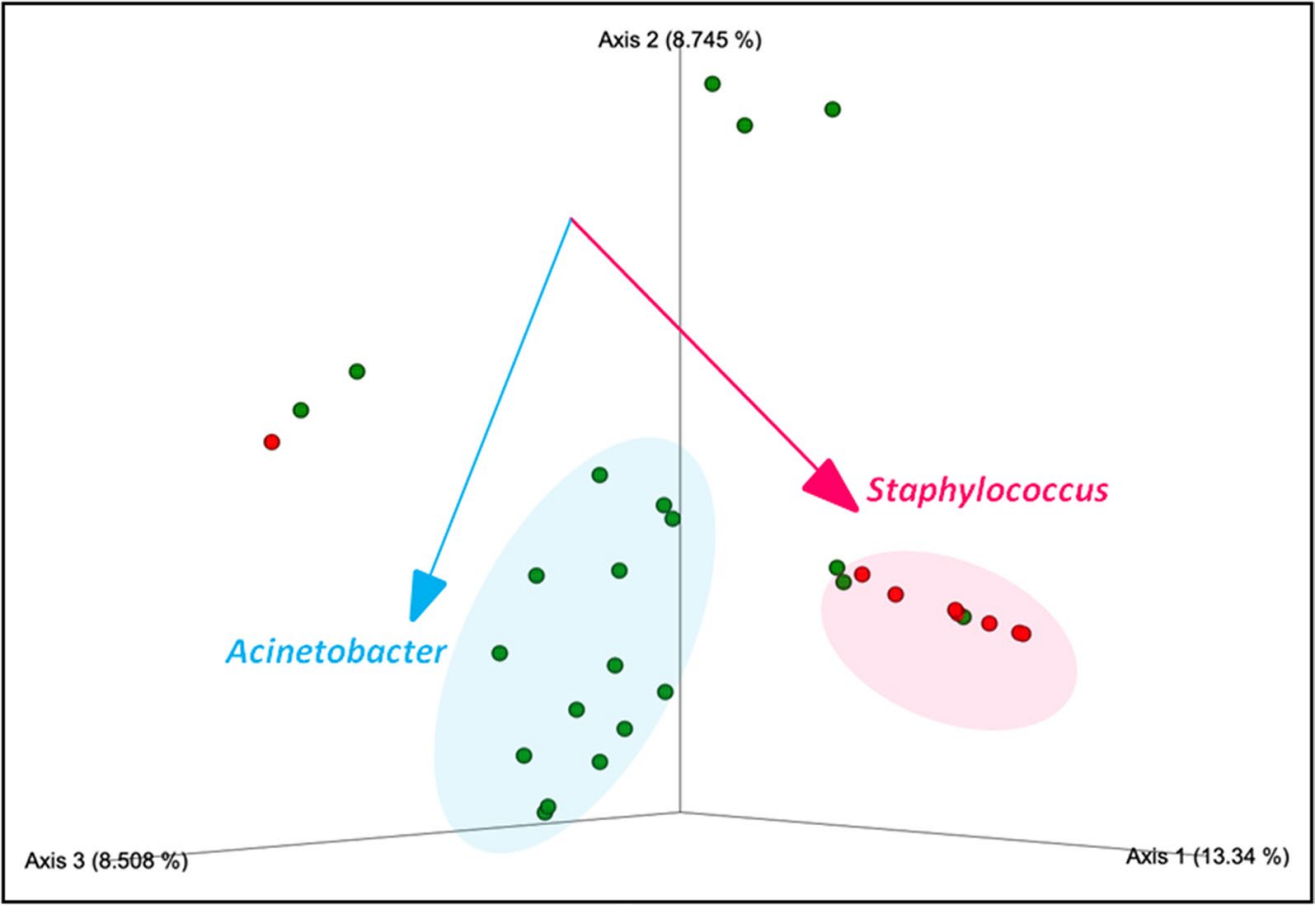
DEICODE robust Aitchison Principal Component Analysis between positive (blue) and negative (red) pneumonia–status groups (PERMANOVA, *pseudo-F* = 7.435*;* *P* = 0.001). Taxa driving the ordination space are indicated by the vectors

Robust Aitchison Principal Component Analysis produced by DEICODE carried out on samples collected at T7 indicated that the microorganisms driving difference (PERMANOVA, *pseudo-F* = 7.435*;* *P* = 0.001) between sampled groups were *Staphylococcus* and *Acinetobacter* (Fig. 7).

These results are consistent with the microbiological results obtained from BAL cultures, which showed pneumonia caused by these two bacterial genera among patients included in the present study (Table 4).

## Discussion

The core finding of this study on lung microbiota in mechanically ventilated traumatic brain injured patients is:

- Specialized nutrition did not display a significant role in preventing VAP and was associated with no significant differences in microbiota composition.
- The analysis of BAL microbiota on patients who developed VAP post-ICU admission showed that at T0 and T7 there were different composition of BAL microbiota in terms of beta diversity. The microorganisms driving difference were *Staphylococcus* and *Acinetobacter.*

It is important to remember that lung microbial community is dynamically renewed and replaced in healthy people, and the majority of microbes involved in these processes belong to four phyla: *Bacteroidetes*, *Firmicutes*, *Proteobacteria*, and *Actinobacteria* [26]. Accordingly, our patients considered as a whole showed the same Phyla, with a prevalence of Firmicutes and Proteobacteria, either at ICU admission and 7 days later. No differences were observed between smokers and no smokers, confirming that smoking does not have a key role in determining modifications of commensal composition of the lower respiratory tract as previously shown [26].

Among the most abundant Genera, we confirmed *Prevotella* and *Streptococcus* at admission in ICU as already described in healthy people and in critically ill patients without ARDS [25, 27]. In the literature, a higher abundance of gut-associated species belonging to Enterobacteriaceae Family, particularly Escherichia coli, Enterobacter spp. and Klebsiella pneumoniae were found to be more abundant in the lung microbiota of patients with ARDS when compared with critically ill patients without ARDS [25]. A recent systematic review showed that inflammation, infections or mortality were significantly lower only in the ICU patients receiving fish oil-derived omega-3 [28], while specialized diets supplemented with arginine with/without additional glutamine or fish oil do not appear to offer an advantage over standard enteral formulas in ICU, trauma and burn patients [29]. In our study, the mortality rate was almost two times higher in the control group than in the specialized nutrition group (47 vs. 25%), but the difference did not reach the statistical significance cutoff value (0.05). This result is most likely because of the small sample size.

Our data showed a higher abundance of members of the Enterobacteriaceae Family, including Escherichia/Shigella, in the specialized nutrition group at T7, while in the standard nutrition group we found higher abundance of Staphylococcus at T7. These results might provide indirect support that different diets, particularly specialized nutrition, may play a role in gut-lung translocation of bacteria thus contributing to the pathogenesis of lung injury in critically ill patients.

Among factors impacting on commensal bacteria growth and survival, nutrients availability and abundance and activation state of host immune cells are involved [30, 31]. Several nutrition strategies are currently being used, showing variable results on beneficial effects on gut microbiota in critically ill patients but to our knowledge no data are available on pulmonary microbiota [25, 28]. Herein, our data showed no differences in alpha and beta diversity of lung microbiota when patients were treated with standard or specialized nutrition. However, we could argue that adaptation of gut microbiota to dietary changes needs a longer time than five days from the start of enteral nutrition and its modulation cannot be so immediate especially in the lungs. Moreover, another factor which might have influenced the observed results is the small sample size; therefore, additional studies would be required to show this correlation.

To our knowledge, our study is the first to show that variation in beta diversity of lung microbiota is correlated to respiratory infections in TBI patients in ICU. This key result is compatible with findings in non-ICU lung microbiota where dysbiosis is a risk factor for several pulmonary disease [25, 27].

During the first week, no patient developed VAP. These data could be explained by the increased clinician’s attention to the employment of effective prevention strategies discussed in the previous narrative review by our group [14].

In our study, a late tracheostomy was performed. Tracheostomy bypasses normal respiratory defense mechanisms, such as oropharynx and cilia, which contribute to the occurrence of VAP. However, the evidence on the advantages of early over late tracheostomy as risk factor for VAP is still debated [32, 33].

Based on the evidence that a group of patients developed VAP during their hospitalization, we retrospectively carried out the alpha and beta diversity analysis on the BAL samples at T0 and T7 in VAP vs no VAP groups. Data on mechanically ventilated critically ill patients with VAP and sepsis demonstrated that reduced microbial diversity in lower respiratory tract reflects high illness severity and are associated with mortality [34].

Interestingly, although we did not observe differences in alpha diversity between the VAP and non-VAP patients, we found significant differences in beta diversity either at admission and seven days after ICU admission between the two groups, suggesting that different structures of BAL microbiota might be able to induce pulmonary infections during hospitalization. These results are consistent with recent studies highlighting the role of the commensal microbes of the respiratory tract in the development of pneumonia [35]. Indeed, there is an increasing awareness that microbes do not act in isolation, rather infections can be the result of complex microbe-host and microbe-microbe interactions able to influence both host susceptibilities to pathogens and severity of infection. In addition, the lower airway microbiota as well as the gut microbiota exert significant effects on host immune response by modulating the immune system, gene expression and bacterial reproduction, with significant effects on the microbial composition and pathogens growth [8]. It is likely that the lung microbiota, as well as gut microbiota, stimulates and shapes the host immune system through changes in its microbial composition and production of bacterial metabolites which can regulate the mucosa immunological tone and host susceptibility to infection. Dysbiosis may alter the well-balanced complex microbial community increasing inflammation and leading to dominance of pathogens with consequent lung injury [35]. Whereas antibiotic administration may have impact on the dysbiosis, the role of antibiotics on upper airways microbiota is not fully understood [34, 35]. A longitudinal analysis of the dynamics of respiratory tract microbiota in critically ill patients highlights the changes occurring in the microbial composition, basically represented by decrease in diversity and dominance of a single taxon over multiple time points [36].

Our data showed that *Staphylococcus* and *Acinetobacter* Genera were higher in the ICU cohort which developed pulmonary infection over two weeks from admission. In those patients, the same genera were identified in the BAL samples by culture method carried out for clinical diagnosis of pneumonia. Six out of the ten patients who developed pneumonia exhibited the presence of *Staphylococcus* and *Acinetobacter*, either alone or with different pathogens identified by cultural method. However, cultural method also identified other pathogens, i.e., *Klebsiella* and *Pseudomonas* in some of the patients who developed pneumonia. Overall, the 16S analysis confirmed the presence of the bacterial genera identified by cultural methods, even though with variable relative abundances. However, only the Genera *Staphylococcus and Acinetobacter* were significantly associated with the differences in the BAL microbiota structure observed between VAP and no VAP groups. Among the genera isolated by cultural methods, only Klebsiella was not confirmed by 16S analysis in the patients who developed pneumonia. This result can be due to the fact that the two analyses, 16S characterization and bacteria cultural tests, were carried out on different samples, collected in different time points. Overall, these data on one hand suggest that the 16S molecular characterization technique can be considered as a sensitive potential diagnostic tool, able to identify the presence of specific pathogens even before the development of the clinical disease; on the other hand, it might be insufficiently accurate to discriminate culture positivity. This observation is consistent with previous reports, showing that untargeted 16S rRNA pathogen detection in lung microbiota may be limited by false positive and negative results. The higher abundance of *Acinetobacter* found in this study in patients who developed pulmonary infection is consistent with dynamics of lung microbiota observed in ICU patients with chronic obstructive pulmonary disease and community acquired pneumoniae [37].

Indeed, these authors found an increased relative abundance of the *Acinetobacter* genus and the *A. baumannii* complex species over time in patients with failed weaning and suggested a possible useful predictive role of this genus on clinical outcomes in critically ill patients.

A limit of our study, besides the small sample size, is that the microbiota analysis was designed only for the first week in ICU. A future study will include samples collection over the first seven days to evaluate the longitudinal microbiota taxonomic profile.

However, the link between changes in the lung microbiota structure and subsequent occurrence of VAP highlights the importance of studying lung microbiota at the ICU admission. The lung microbiota is an understudied source of clinical variation in critical illness and represents a novel therapeutic target for the prevention and treatment of acute respiratory failure.

## Conclusion

In conclusion, although further research is needed to fully understand the complex interplay among nutrition, infections and microbiota composition, our data suggest that TBI patients who developed VAP during ICU stay have different structures of BAL microbiota either at admission and at 7 days post-ICU admission, while no correlation has been observed between different enteral formulas and microbiota composition in terms of richness and evenness. These findings suggest that targeting the lung microbiota may be a promising approach for preventing infections in critically ill patients.

## Data Availability

The datasets used and analyzed during the current study are available from the corresponding author upon reasonable request.

## Abbreviations

ICU: Intensive care unit
TBI: Traumatic brain injury
VAP: Ventilator-associated pneumonia
BAL: Bronco Alveolar Lavage
SAPS II: Simplified Acute Physiology Score
APACHES II: Acute Physiologic Assessment and Chronic Health Evaluation Score
ARDS: Acute respiratory distress syndrome
BMI: Body mass index
PCA: Principal component analysis
ASVs: Amplicon Sequence Variants
